# Cardiovascular and Diabetes Diseases Classification Using Ensemble Stacking Classifiers with SVM as a Meta Classifier

**DOI:** 10.3390/diagnostics12112595

**Published:** 2022-10-26

**Authors:** Asfandyar Khan, Abdullah Khan, Muhammad Muntazir Khan, Kamran Farid, Muhammad Mansoor Alam, Mazliham Bin Mohd Su’ud

**Affiliations:** 1 Institute of Computer Science and Information Technology, ICS/IT FMCS the University of Agriculture, Peshawar 25130, Pakistan; 2 Faculty of Computing, Riphah International University, Islamabad 46000, Pakistan; 3 Faculty of Computing and Informatics, Multimedia University, Cyberjaya 63100, Malaysia

**Keywords:** KNN, Naive Bayes, decision tree, diabetes disease, stacking classifier, cardiovascular disease, meta-classifier, coronary heart diseases

## Abstract

Cardiovascular disease includes coronary artery diseases (CAD), which include angina and myocardial infarction (commonly known as a heart attack), and coronary heart diseases (CHD), which are marked by the buildup of a waxy material called plaque inside the coronary arteries. Heart attacks are still the main cause of death worldwide, and if not treated right they have the potential to cause major health problems, such as diabetes. If ignored, diabetes can result in a variety of health problems, including heart disease, stroke, blindness, and kidney failure. Machine learning methods can be used to identify and diagnose diabetes and other illnesses. Diabetes and cardiovascular disease both can be diagnosed using several classifier types. Naive Bayes, K-Nearest neighbor (KNN), linear regression, decision trees (DT), and support vector machines (SVM) were among the classifiers employed, although all of these models had poor accuracy. Therefore, due to a lack of significant effort and poor accuracy, new research is required to diagnose diabetes and cardiovascular disease. This study developed an ensemble approach called “Stacking Classifier” in order to improve the performance of integrated flexible individual classifiers and decrease the likelihood of misclassifying a single instance. Naive Bayes, KNN, Linear Discriminant Analysis (LDA), and Decision Tree (DT) are just a few of the classifiers used in this study. As a meta-classifier, Random Forest and SVM are used. The suggested stacking classifier obtains a superior accuracy of 0.9735 percent when compared to current models for diagnosing diabetes, such as Naive Bayes, KNN, DT, and LDA, which are 0.7646 percent, 0.7460 percent, 0.7857 percent, and 0.7735 percent, respectively. Furthermore, for cardiovascular disease, when compared to current models such as KNN, NB, DT, LDA, and SVM, which are 0.8377 percent, 0.8256 percent, 0.8426 percent, 0.8523 percent, and 0.8472 percent, respectively, the suggested stacking classifier performed better and obtained a higher accuracy of 0.8871 percent.

## 1. Introduction

Cardiovascular disease (CVD) leads to artery damage in organs such as the kidneys, heart, eyes, and brain. Therefore, it poses a threat to people’s health [1]. Diabetes is a harmful illness and can result in serious health problems such as kidney failure, stroke, heart disease, and blindness [2]. Additionally, even among young individuals, CVD is one of the leading causes of mortality in many industrialized and developing nations worldwide.

Cardiovascular illness requires careful handling due to its intricacy. If not, heart health might suffer, and sudden death may occur [2]. For a classification of various forms of metabolic illnesses, medical science and statistical viewpoints are used. For data study and cardiac disease prediction, data analysis with classification is crucial [3]. 

A number of machine learning methods can be used to examine this data. In the field of cardiovascular detection and classification, machine learning (ML), a subset of artificial intelligence (AI), is being used more and more. In essence, it describes how computers interpret data and categorize or decide on a job, whether or not human oversight is involved. Models that take in input data (such as photos or text) and predict results using a combination of mathematical optimization and statistical analysis are the foundation of machine learning (ML) theory (e.g., favorable, unfavorable, or neutral). Numerous ML techniques have been used to model everyday activities [4]. A variety of data analysis and neural network techniques have been employed to assess the severity of heart illness in patients [5]. This data is analyzed by healthcare professionals to help them choose the best diagnostic approach. Medical data mining with categorization algorithms provides therapeutic support through analysis. It assesses tools for categorizing patients’ risk of heart disease [6].

The K-Nearest Neighbor (KNN), DT, Genetic Algorithm (GA), and Naive Bayes (NB) algorithms are a few of these methods used to identify the severity of sickness [5]. Several research studies have been conducted and machine learning models have been used in order to categorize and predict heart disease diagnoses. In the medical field, artificial neural network (ANNs) were created to obtain the highest prediction accuracy possible [7,8]. Through the use of a back propagation multilayer perceptron, ANNs are employed in [8] to predict cardiac illness. When the results are compared with those of previously published models in the same field, it is discovered that they have greatly improved. In [9], data from the UCI laboratory on patients with cardiac illness are used to find patterns using ANN, DT, Support Vector Machines (SVMs), and Naive Bayes. Different algorithms’ effectiveness and precision are contrasted. The accuracy of the suggested hybrid strategy, which is 86.8%, is equivalent to that of other approaches that are currently in use. Golande et al., looked at several machine learning techniques that may be applied to categorize heart illness. The accuracy of the DT, KNN, and K-Means algorithms, which might be used for classification, has been investigated [10]. This study shows that DTs obtain the highest accuracy and that they may be made more effective by combining a variety of techniques and adjusting certain parameters. Data mining techniques and the MapReduce algorithm were merged in a system created by Nagamani et al. [11]. The accuracy gained in this study was better than the accuracy obtained using a standard fuzzy artificial neural network for the 45 occurrences in the testing set. The accuracy of the approach was improved in this instance due to the use of dynamic schema and linear scaling. A machine learning model created by Alotaibi analyses five different approaches [12]. When compared to MATLAB and Weka, a quick miner delivered a higher degree of accuracy.

The accuracy of the classification algorithms DT, LR, NB, and SVM was compared in this study. The most accurate algorithm was the one using decision trees. Research by Thomas and Princy compared several classification algorithms that are used to forecast cardiac disease. Naive Bayes, KNN, DT, and Neural Network were the classification techniques used and the accuracy of the classifiers was assessed across a variety of attribute counts [3]. In order to forecast heart illness, Lutimath et al., employed SVM and Naive Bayes classification. The Root Mean Square Error, the Sum of Squared Error, and the Mean Absolute Error are the performance measures used in the study. In addition, SVM has been demonstrated to perform better than Naive Bayes in terms of accuracy [13].

Hossen et al., in [14], conducted a survey which is divided into three sections: classification and data mining techniques for CVD, machine learning models for CVD, and deep learning models for CVD prediction. This survey also compiles and reports the performance metrics used for reporting accuracy, the dataset utilized for prediction and classification, and the tools used for each category of these approaches. Similarly, SVM, MLP, Random Forest, Logistic Regression, and Decision Tree were some of the algorithms used by Sharma et al. [15]. Patients’ diabetes can be predicted more accurately using the PIMA dataset. Another study that used the PIMA dataset produced significant findings for Naive Bayes [16]. Kuchi et al. [17] attained a 95.4% accuracy using the stacking method. According to Kavakiotis et al. [18], the diabetes diagnostic problem requires further investigation. The accuracy of diabetic illness prediction can be improved by combining several classifiers.

As a result, this study overcomes the limitations of diabetes classification by combining different classifiers for high accuracy. The primary goal of the suggested system was to create a computer-aided diagnostic system after carefully examining the aforementioned research. For both the cardiovascular illnesses dataset and the diabetic diseases dataset, we examined the accuracy, precision, recall, and F1-scores of all classification methods. The following are the primary contributions of this study:

- The study used two different stacking classifier models for the cardiovascular and diabetes disease classification.
- For the cardiovascular diseases dataset, four algorithms such as KNN, NB, DT, and LDA are used as base algorithms, and SVM is used as a meta-classifier.
- Similarly for the diabetes diseases dataset, four algorithms such as KNN, NB, DT, and LDA are used as base algorithms and RF is used as a meta-classifier.
- The performance of all these models are compared in terms of accuracy, precision, recall, and F-measure with individual models such as KNN, NB, DT, SVM and LDA.

Three sections make up the remaining parts of this paper. Section 2 presents the approach and techniques, Section 3 presents the findings and analysis, and Section 5 presents the conclusion and future directions.

## 2. Materials and Methods

This section provides a detailed explanation of the proposed stacking method, data collection, data exploration, and the stacking classifier. The following is a description of the stacking classifier:

### 2.1. Stacking Classifier

**Figure 1:**
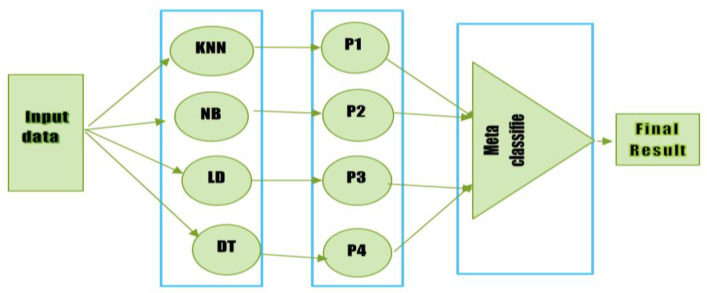
Diagram of a stacking classifier.

An ensemble learning method called stacking enables a meta-classifier to mix several classification models. A meta-classifier is used to classify the output received from different classifiers (sometimes referred to as level one or base classifiers). Any classifier may be used as a meta-classifier to improve performance. The training of four different classifiers is shown in Figure 1. The outputs of the base classifiers are merged and used to train the meta-classifier, which generates the final prediction. This stack uses four classifiers, each of which was trained separately. After that, they stack their predictions to train the meta-classifier. Three rules serve as the foundation for the stacking classifier.

- In the first phase, the input data is passed to the base classifiers individually to generate the output.
- Then the individual output of each classifier is combined and passed as input to the meta-classifier.
- Finally, the meta-classifier is trained on the combined data (received from the base classifiers) to generate a final prediction.

### 2.2. Dataset Collection

**Figure 2:**
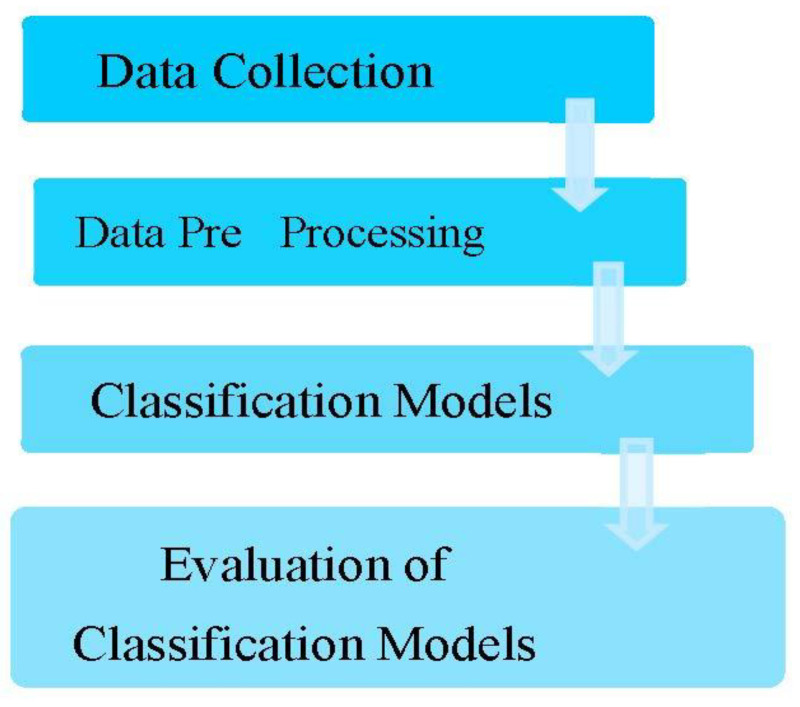
General workflow of prediction systems.

In this research, two datasets were used, one for cardiovascular disease and one for diabetes disease. The diabetes dataset was retrieved from the (https://www.kaggle.com/johndasilva/diabetes accessed on 9 May 1990) website. The data was taken from the hospital Frankfurt, Germany. The data in the diabetes dataset were records from 2000 females in experimental research. Table 1 displays the dataset characteristics where “1” is seen as positive and “0” as negative. Furthermore, “1” stands for a diabetic patient, whereas “0” is a patient with no diabetes. In the diabetes dataset, there are nine characteristics, including Pregnancies, Glucose, Blood Pressure, Skin Thickness, Insulin, BMI, Diabetes Pedigree Function, Age, and Outcome. The cardiovascular disease data set is collated from the given link: https://www.kaggle.com/datasets/christofel04/cardiovascular-study-dataset-predict-heart-disea accessed on 3 October 2020. This dataset has 4000 instances overall and 15 characteristics. Table 2 below provides more information on the various properties and classes. Giving the typical process of prediction systems, Figure 2 describes the categorization workflow for the various research processes.

### 2.3. Data Exploration

**Figure 3:**
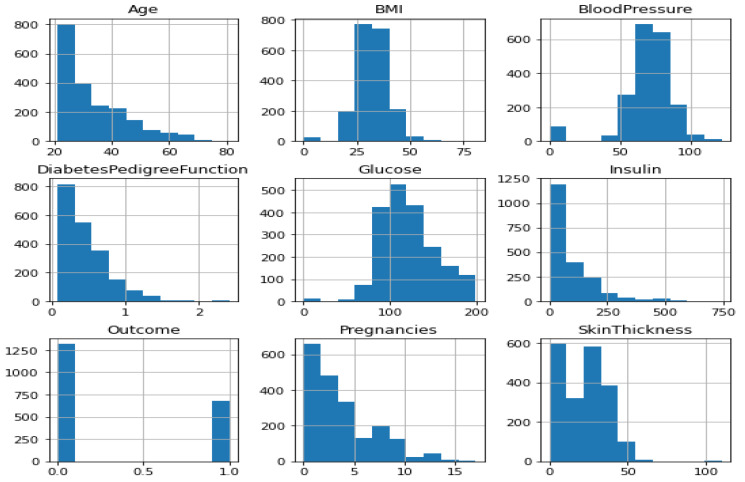
Data distribution of diabetes.

**Figure 4:**
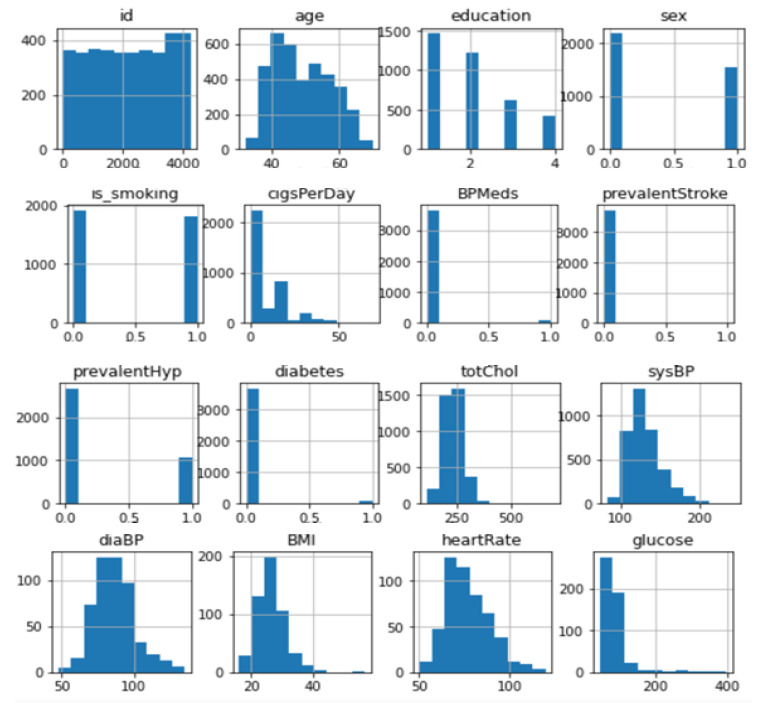
Data distribution of cardiovascular disease.

The dataset was converted into a machine-readable format after being downloaded in raw form. Any algorithm’s accuracy depends on the type and quantity of data it has access to. The original data was cleaned up since it was unreliable and noisy before being fed into the classifier for prediction. Because of this, pre-processing methods are essential for raising data quality, which also raises classification precision. The diabetes dataset has several missing variables that cause uncertainty and provide incorrect conclusions. The recommended research data cleaning process is crucial because of the uncertainty. During the data cleaning process, missing values are handled, noise is reduced, and dataset inconsistencies are fixed. Table 3 displays the diabetes dataset. The diabetes dataset consists of 2000 rows and 9 columns. The “outcome” and goal characteristic, represented by the final column, show whether or not the patient has diabetes. Additionally, there are only two numbers in the result column: 1 for diabetes and 0 for non-diabetic. The Panda “Matplotlib” developed library is used to display and recognize the feature distribution seen in Figure 3 and Figure 4.

## 3. Proposed Stacking Model Algorithm

**Figure 5:**
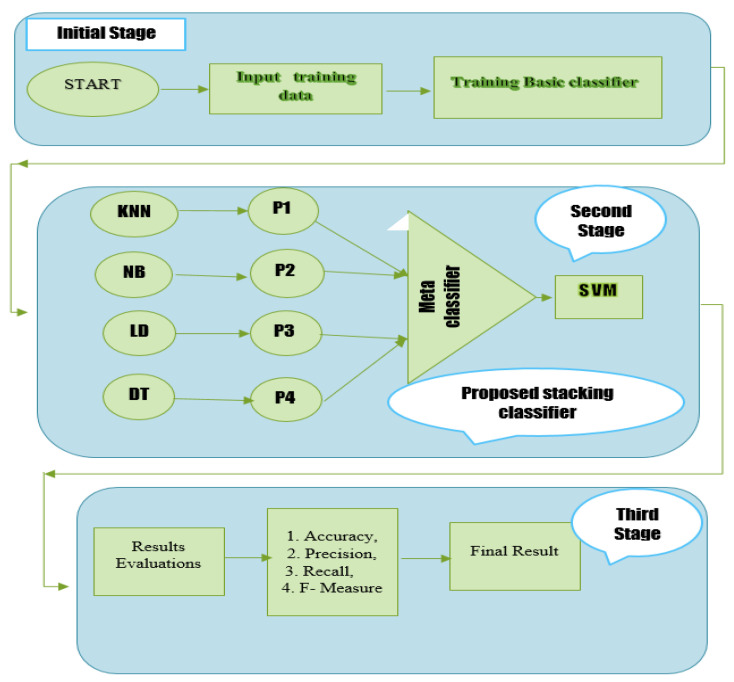
Proposed stacking model process flow chart.

KNN, Linear Discriminant Analysis, Decision Tree, and K-Nearest Neighbor Naive Bayes are used to train the training component dataset. The extra characteristics produced by the original base proposed model classifier are trained using the meta-classifier in order to acquire them. Two alternative methodologies were employed in this study to conduct the investigation. First, this study used the Random Forest as a meta-classifier to group the final diabetic illness prediction. Then, the SVM technique was also employed in a second strategy to categorize the final prediction for the cardiovascular disorders dataset using a meta-classifier. Figure 5 displays the whole flow diagram of our proposed stacking paradigm. There are three steps that must be taken while implementing this proposed paradigm. The first training dataset is constructed and trained using KNN, NB, RF, and DT in this phase. The 70% training dataset is used to train the KNN, Naive Bayes Model, Linear Discriminant Analysis (LDA), and Decision Tree. After the four models (KNN, NB, LDA, and DT) have been trained in the first step, the predictions of each model are obtained. A fresh dataset is constructed in the third stage using the predictions from the first basic classifiers (KNN, NB, LDA, and DT). The first step of the base four classifiers will cause the new dataset to have four dimensions. A second-level classifier called a meta-classifier is used on the first-stage dataset. In this study, a meta-classifier called Random Forest and SVM were used. This research will also train and analyze each model separately to evaluate the effectiveness and accuracy of the suggested stacking model. The suggested stacking model’s performance is also contrasted with that of individual classifiers such as KNN, Naive Bayes, Linear Discriminant Analysis, and Decision Tree in terms of Recall, Precision, and F-Measure. The proposed stacking model architecture is presented in Figure 5. In Algorithm 1, the suggested stacking model is presented.
**Algorithm 1:** The Proposed Stacking Model1.Start2.Input data: Input training dataset $X={\left\{x_{i},y_{i}\right\}}_{i=1}^{m}$3.Output data: Stacking classifier output result.4.Pre-process the input data5.Set all the model architecture according to the input data6.Train all the base classifiers7.for t = 1 to T   do8.learn $output_{t}$ based on X9.end for10.Generate new dataset of the base classifiers11.For i=1 to m do$X_{bc}=\left\{{x^{\prime }}_{bc}\;,y_{i}\right\}$  where ${x^{\prime }}_{bc}$= $\left\{output_{1\left(x_{i}\right)},\ldots \ldots output_{T\left(x_{i}\right)}\;\right\}$12.end for13.Learn SVM as a mate classifier in the proposed model14.Learn $y_{i}$ base on $X_{bc}$15.Generate the final output $Y_{i}$16.End

### 3.1. Performance Parameters

The Python version 3.6 is utilized in this study for experimental work, with the following parameters given below.

#### 3.1.1. Recall

Recall is the summation of all correctly identified positive values, which is divided by the total number of true positive and false negative values. “True Positive Rate” measures mean positive factors which are identified correctly. The high recall specifies the correctly diagnosed cases.

(1) $$Recall=\frac{TP}{TP+FN}$$

#### 3.1.2. Precision

Precision is calculated from all correctly identified positive values, which is divided by the total number of true positive and false positive values.

(2) $$Precision=\frac{TP}{TP+FP}$$

#### 3.1.3. F-Measure

F-Measure is calculated from recall and precision as given below:

(3) $$F\;Measure=\frac{2\;*\;Recall\;*\;Precision}{Recall+Precision}$$

#### 3.1.4. Accuracy

Accuracy indicates how comfortable the model is with detecting the positive and negative classes.

(4) $$Accuracy=\frac{TP+TN}{TP+TN+FP+FN}$$

where *TP*: is true positivity, *TN*: is true negative, *FP*: is false positive, and *FN*: is false negative.

## 4. Results and Discussion

Further evaluations and validations of the suggested model’s performance were conducted in terms of recall, precision, f-measure, and accuracy, and the proposed stacking classifier’s performance is compared to that of the KNN, NB, LDA, DT, and SVM algorithms. In this work, two datasets related to diabetic disorders and cardiovascular diseases are utilized to evaluate the effectiveness of the proposed stacking classifiers. The model’s performance was evaluated using accuracy, loss, precision, f-measure, and recall. The models were used with training data as well as testing data. A 70:30 split of the data was made for training and testing purposes.

### 4.1. Preliminaries

The studies were carried out using an 8 GB RAM, 2.0 GHz Intel Core i5 CPU. Windows 10 served as the operating system. For all datasets, the model was tested and trained using the Keras Python package. In order to explore the suggested stacking classifiers, KNN, NB, LDA, DT, and SVM algorithms are compared in terms of recall, precision, f-measure, and accuracy.

### 4.2. Experimental Result

Several tests were carried out on five models, which were executed using various metrics including accuracy, recall, precision, and f-measure. The following is a list of models that are used in simulation: 1. Proposed Stacking Classifier, 2. KNN, 3. Naive Bayes, 4. Linear Discriminant Analysis (LDA), and 5. Decision Tree.

### 4.3. Performance Evaluations on Cardiovascular Disease

**Figure 6:**
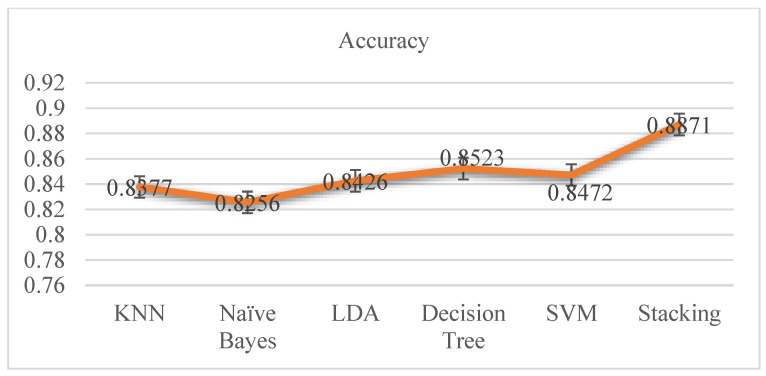
Accuracy Graphs of Classifiers on Cardiovascular Disease.

The accuracy differences of KNN, Naive Bayes, LDA, decision tree, and stacking classifiers are shown in Table 4 and Figure 6. From Table 4, it is seen that the KNN model attains 0.8377 percent accuracy and the Naive Bayes model gets 0.8256 percent accuracy. Figure 6 depicts the accuracy line from KNN to NB utilizing KNN and SVM to stack classifiers. LDA performed admirably, with a respectable accuracy of 0.8426 percent. Additionally, SVM and Decision Tree both have accuracy ratings of 0.8472 and 0.8523 percent, respectively. Finally, the proposed stacking classifier has an accuracy rate of 0.8871 percent. As a result, this study finds that integrated classifiers perform better than separate classifiers. The proposed stacking model, which combines the four classifiers listed above, has a higher accuracy of 0.8871 percent than the other classifiers.

**Figure 7:**
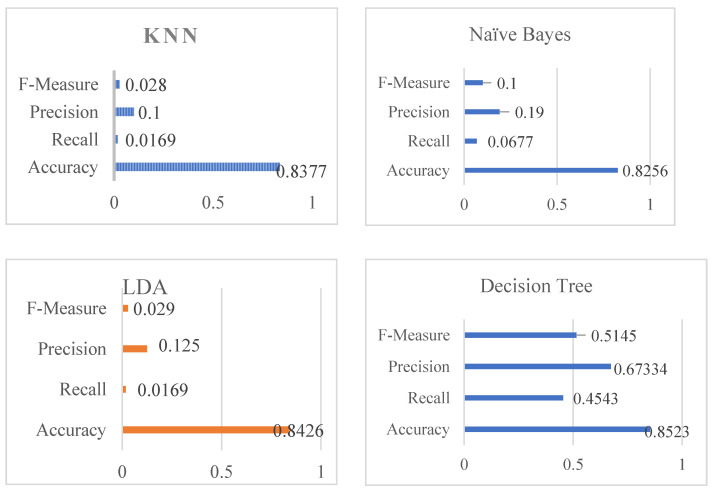

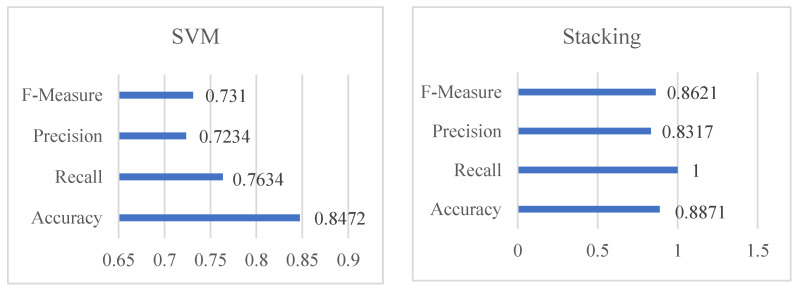
Accuracy, recall, precision, and f-measure graphs on cardiovascular disease.

**Figure 8:**
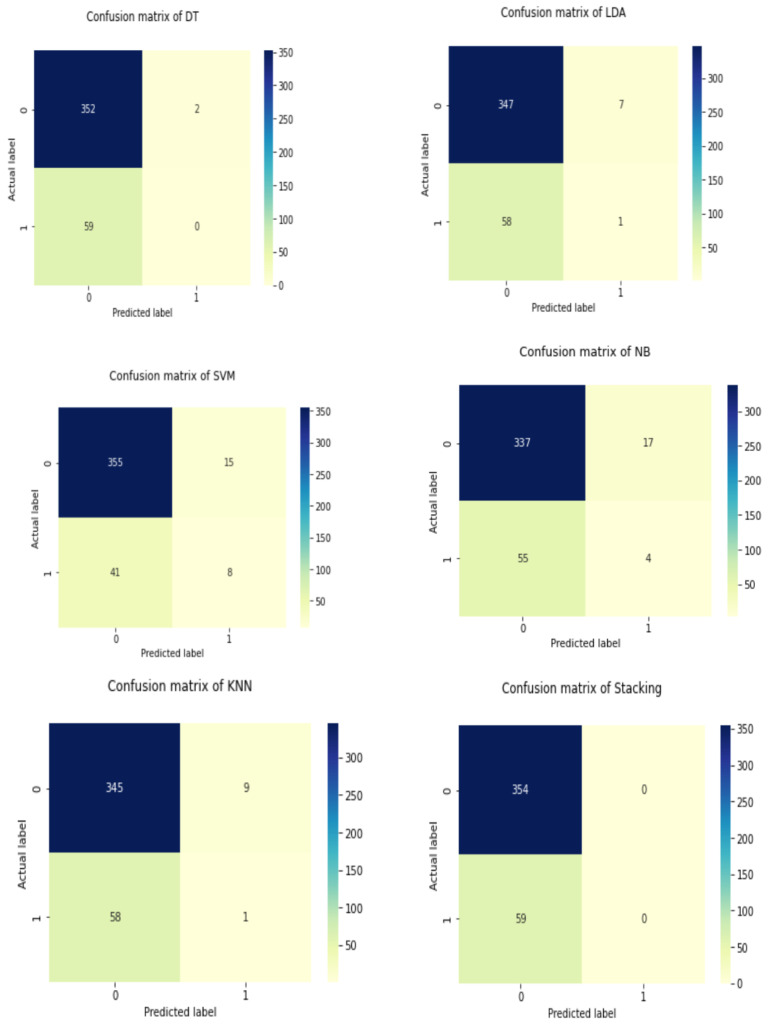
Confusion matrix of the used models on cardiovascular disease.

Table 4 shows the overall performance of the stacking classifier, KNN, Naive Bayes, Linear Discriminant Analysis, and Decision Tree in terms of Accuracy, Recall, Precision, and F-Measure. Table 4 shows that KNN obtained impressive results, including 0.028 percent f-measure, 0.100 percent precision, 0.0169 percent recall, and 0.100 percent accuracy. Following the analysis, Naive Bayes produces a significant result of 0.8256 percent accuracy, recall of 0.0677 percent, precision of 0.190 percent, and F-measure of 0.100 percent. The accuracy score of Linear Discriminant Analysis (henceforth referred to as LDA) was determined to be 0.8426 percent, 0.0169 percent recall, 0.125 percent precision, and finally 0.029 percent f-measure using 80 percent training and 20 percent testing data. The DT was found to be 0.8523 percent for accuracy, 0.4543 percent recall, 0.67334 percent precision, and lastly 0.5145 percent for f-measure, whereas SVM was found to have an accuracy of 0.8472 percent, a recall of 0.7634 percent, a precision of 0.7234 percent, and an f-measure of 0.731%. The stacking classifier’ has achieved an accuracy, recall, precision, and F-measure of 0.8871 percent, 0.8871 percent, 0.8317 percent, and 0.8621 percent, respectively. Table 4 and Figure 7 illustrate the stacking classifier’s overall performance on cardiovascular disease. The confusion matrix of the model used to describe cardiovascular illness is shown in Figure 8.

### 4.4. Performance Evaluations on Diabetes Disease Dataset

**Figure 9:**
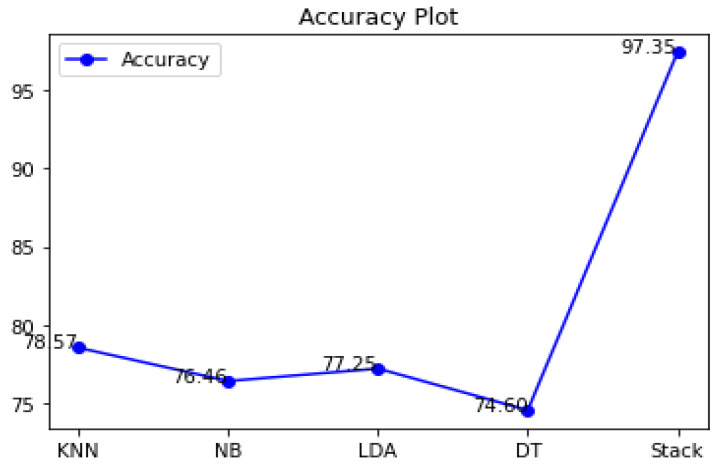
Accuracy graphs of classifiers.

The accuracy differences of KNN, Naive Bayes, LDA, decision tree, and stacking classifiers are shown in Table 5 and Figure 9. Table 5 shows the KNN model obtaining 0.7857 percent accuracy and the Naive Bayes model getting 0.7646 percent accuracy. Figure 8 depicts the accuracy line from KNN to NB utilizing KNN and DT to stack classifiers. LDA performed admirably, with a respectable accuracy of 0.7725 percent. Additionally, Decision Tree has an accuracy score of 0.7460 percent. Finally, the proposed stacking classifier has an accuracy rate of 0.9735 percent. As a result, this study finds that integrated classifiers perform better than separate classifiers. The recommended stacking model, which combines the four classifiers listed above, has a higher accuracy of 0.9735 percent than the other classifiers.

**Figure 10:**
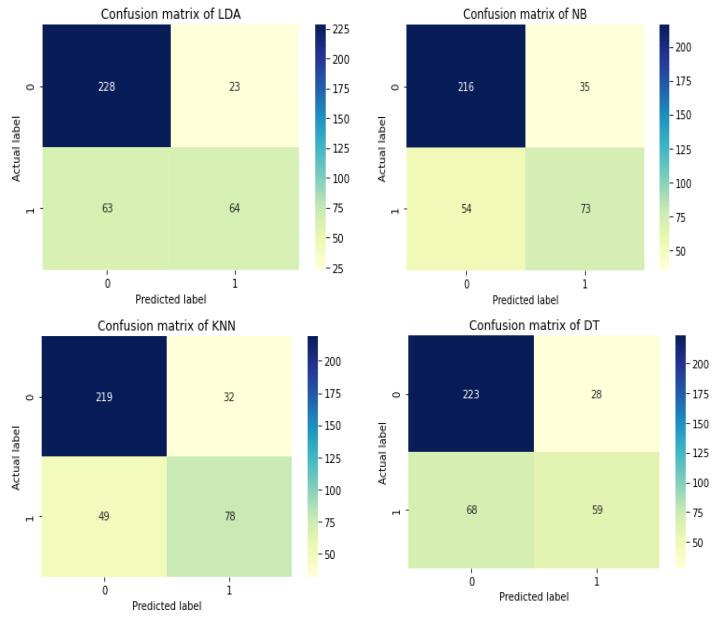
Confusion matrix of the used models on the diabetes dataset.

**Figure 11:**
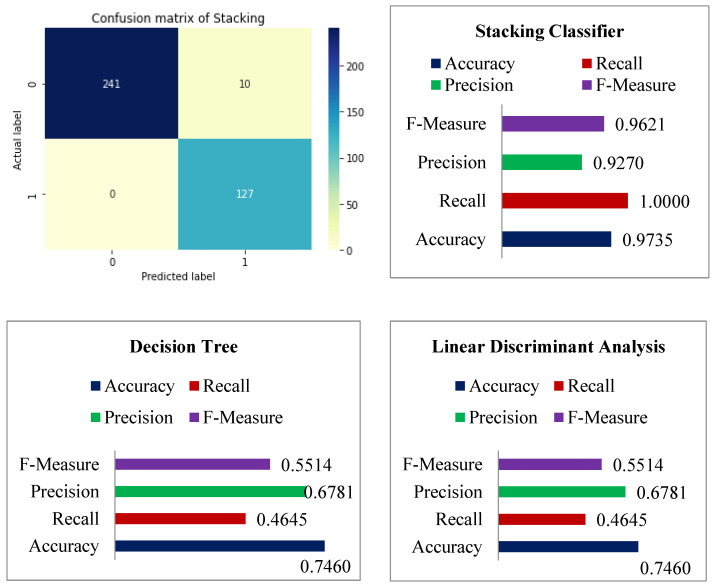

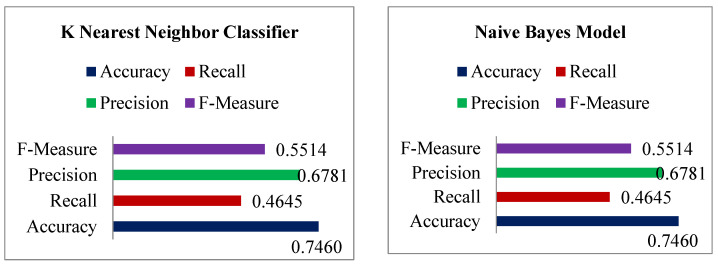
Accuracy, Recall, Precision, and F-measure performances of used models on diabetes dataset.

In terms of accuracy, recall, precision, and F-Measure, Table 5 displays the overall performance of the stacking classifier, KNN, Naive Bayes, Linear Discriminant Analysis, and Decision Tree. Table 5 demonstrates that KNN achieved remarkable results, such as 0.7857 percent accuracy, 0.6141 percent recall, 0.7091 percent precision, and lastly, 0.6582 percent f-measure. Following the examination, Naive Bayes achieves a notable result of 0.7646 percent accuracy with a recall of 0.5748 percent, while precision is 0.6759 percent and 0.6213 percent for f-measure. With 80 percent training and 20 percent testing data, the accuracy score of LDA was found to be 0.5981 percent for f-measure, 0.7725 percent accuracy, 0.5039 percent recall, and lastly, 0.7356 percent precision. The DT was found to be 0.5514 percent for f-measure, 0.7460 percent accuracy, 0.4646 percent recall, and lastly, 0.7356 percent precision. The accuracy of the stacking classifier was found to be 0.9621 percent for f-measure, 0.9735 percent accuracy, 1.00 percent recall, and lastly, 0.9217 percent for precision. Figure 10 shows the confusion matrix of the used model for diabetes disease. Similarly, the total performance of the stacking classifier on diabetes disease is shown in Figure 11.

## 5. Conclusions

In order to predict individuals with diabetes and cardiovascular disease, many studies have been conducted in the past to diagnose diabetes and cardiovascular disease using a single classifier. However, in this study, the diabetic disease and cardiovascular disease classification was done using a stacking classifier approach. Basic classifiers include KNN, Naive Bayes, Linear Discriminant Analysis, and Decision Trees, whereas meta-classifiers include Random Forest and SVM. The proposed model is contrasted with the other cardiovascular disease classifiers, including KNN, Naive Bayes, LDA, SVM, and Decision Tree, in terms of accuracy, recall, precision, and f-measure. The recommended stacking with Random Forest serving as a meta-classifier is compared to the other classifiers such as KNN, Naive Bayes, LDA, and DT on the diagnosis of diabetic illness in a similar manner in terms of accuracy, recall, precision, and f-measure. The accuracy rate of the proposed stacking classifier is 0.8871 percent, while the accuracy of the Naive Bayes model is 0.8256 percent and that of the KNN model is 0.8377 percent. The accuracy of LDA was 0.8426 percent. Additionally, SVM and Decision Tree have accuracy ratings of 0.8472 and 0.8523 percent for cardiovascular illness, respectively. This study shows that on the diabetes dataset, integrated classifiers outperform individual classifiers. The recommended stacking model, which combines the four classifiers listed above, has a higher accuracy of 0.9735 percent than the other classifiers on the diabetes dataset.

## Figures and Tables

**Table 1:** Diabetes dataset.

	Pregnancies	Glucose	Blood Pressure	Skin Thickness	Insulin	BMI	Diabetes Pedigree Function	Age	Outcome
**0**	2	138	62	35	35	33.6	0.127	47	1
**1**	0	84	82	31	31	38.2	0.233	23	0
**2**	0	145	0	0	0	44.2	0.630	31	1
**3**	0	135	68	42	250	42.3	0.365	24	1
**4**	1	139	62	41	480	40.7	0.536	21	0

**Table 2:** Cardiovascular disease dataset.

ID	Age	Education	Sex	Is_Smoking	cigsPerDay	BPMeds	Prevalent Stroke	Prevalent Hyp	Diabetes	totChol	sysBP	diaBP	BMI	Heart Rate	Glucose	Ten Year CHD
0	0	64	2	0	1	3	0	0	0	221	148.0	85.0	25.40	90	80	1
1	36	4	1	0	0	0	0	1	0	212	168.0	98.0	29.77	72	75	0
2	46	1	0	1		10	0	0	0	250	116.0	71.0	20.35	88	94	0
3	50	1	1	1	20	0	0	1	0	233	158.0	88.0	28.26	68	94	1
4	64	1	0	1	30	0	0	0	0	241	136.5	85.0	26.42	70	77	0

**Table 3:** Numerical attributes of patient dataset.

Sr. No	Attribute	Values
1	Number of times pregnant/Germination	[0–17]
2	Plasma Glucose Level	[0–199]
3	Diastolic Blood Pressure	[0–122]
4	Triceps Skin-Fold Thickness	[0–99]
5	2-h Serum Insulin	[0–846]
6	Body Mass Index	[0–67]
7	Diabetes Pedigree Function	[0–2.45]
8	Age	[21–81]
9	Class (Positive or Negative)	[0,1]

**Table 4:** Comparison of classification techniques on cardiovascular disease.

Model	Accuracy	Recall	Precision	F-Measure
**KNN**	0.8377	0.0169	0.100	0.028
**Naive Bayes**	0.8256	0.0677	0.190	0.100
**LDA**	0.8426	0.0169	0.125	0.029
**DT**	0.8523	0.4543	0.67334	0.5145
**SVM**	0.8472	0.7634	0.7234	0.731
**Stacking**	0.8871	0.8871	0.8317	0.8621

**Table 5:** Comparison of classification techniques on diabetes disease dataset.

Model	F-Measure	Accuracy	Recall	Precision
**KNN**	0.6582	0.7857	0.6141	0.7091
**Naive Bayes**	0.6213	0.7646	0.5748	0.6759
**LDA**	0.5981	0.7725	0.5039	0.7356
**DT**	0.5514	0.7460	0.4646	0.6782
**Stacking**	0.9621	0.9735	1.0000	0.9217

## Data Availability

In this research, two datasets were used, one for cardiovascular disease and one for diabetes. The diabetes dataset was retrieved from the (https://www.kaggle.com/johndasilva/diabetes accessed on 9 May 1990) website. The data collection was conducted at the hospital in Frankfurt, Germany. The cardiovascular disease data set is collated from the given link: https://www.kaggle.com/datasets/christofel04/cardiovascular-study-dataset-predict-heart-disea accessed on 3 October 2020.
